# Deep Learning-Based Calibration of a Multi-Point Thin-Film Thermocouple Array for Temperature Field Measurement

**DOI:** 10.3390/s26061956

**Published:** 2026-03-20

**Authors:** Zewang Zhang, Shigui Gong, Jiajie Ye, Chengfei Zhang, Jun Chen, Zhixuan Su, Heng Wang, Zhichun Liu, Zhenyin Hai

**Affiliations:** 1 School of Opto-Electronic and Communication Engineering, Xiamen University of Technology, Xiamen 361005, China; 2 Inner Mongolia Aerospace Power Machinery Testing Institute, Hohhot 010076, China; 3 School of Electronic Engineering, Ocean University of China, Qingdao 266100, China; 4 School of Aerospace Engineering, Xiamen University, Xiamen 361005, China

**Keywords:** multi-point array thermocouple, deep learning, multilayer perceptron, temperature field monitoring

## Abstract

Multi-point array thin-film thermocouples have strong potential for high-precision, wide-range temperature monitoring in applications such as aircraft engine thermal condition assessment and industrial process control. However, conventional single-point thin-film thermocouples cannot satisfy the distributed measurement requirements of large-area temperature fields, and the accuracy of multi-point arrays is often degraded by coupling effects among sensing nodes, which hinders their engineering deployment. In this work, a multi-point array thin-film thermocouple is fabricated via precision welding, and an insulating layer is deposited on the sensor surface using electrospray atomization to establish a multi-point temperature-sensing hardware system. To compensate for coupling-induced deviations, a deep learning–based calibration method is developed: measurements from the array and reference thermocouples are synchronously collected to build the dataset, outliers are removed using the interquartile range (IQR) method, and a three-hidden-layer multilayer perceptron (MLP) is trained for each node independently using the Adam optimizer (learning rate 0.001) with an 8:2 train–test split. Performance is quantified by MAE, MSE, and R^2^, and the results show that the proposed approach markedly reduces measurement errors and improves the accuracy of the array thermocouples, demonstrating reliable performance and practical applicability for precise large-area temperature-field monitoring.

## 1. Introduction

As a fundamental and critical physical parameter in industrial production, aerospace, and environmental monitoring, the accuracy and range of temperature measurements are essential for ensuring the safety, stability, and efficiency of system operations [1,2,3]. In aero-engine thermal state monitoring, real-time acquisition of temperature distribution across key components—such as the combustion chamber and turbine blades—provides vital data for assessing component fatigue, optimizing combustion efficiency, and ensuring flight safety [4,5,6]. In industrial process control, maintaining uniform temperature fields in chemical reactors or metallurgical furnaces is crucial for ensuring product quality and minimizing energy consumption. Likewise, in complex multi-point temperature monitoring environments—such as large data centers and cold-chain logistics warehouses—tracking temperature gradients across wide areas is essential for early fault detection and efficient energy management. As temperature monitoring needs in these sectors shift from single-point, static measurements to multi-point, dynamic, and large-area distributed sensing, conventional temperature measurement technologies are increasingly facing limitations [7,8]. This growing demand highlights the urgent need for innovative solutions to overcome existing bottlenecks in temperature measurement.

In conventional temperature measurement, single-point thin-film thermocouples are widely used for localized monitoring due to their simple structure and low cost. However, their “one-to-one” measurement principle limits them to capturing temperature data from a single location within the area of interest. To monitor large-area temperature fields, a significant number of individual thermocouples must be deployed. This increases system complexity and hardware costs, while complicating the synchronization and integrated analysis of multi-point temperature data. As a result, these traditional approaches struggle to meet the modern industry’s demand for high-precision, wide-range temperature field monitoring. The emergence of multi-point array thin-film thermocouples offers a promising solution to these challenges [9,10,11]. By integrating multiple sensing nodes onto a single substrate and applying a high-temperature insulating layer to its surface, this technology enables synchronous temperature measurement across multiple locations. This provides effective coverage of large areas and overcomes the limitations of “scattered measurement points and unsynchronized data” inherent in conventional single-point thin-film thermocouples. As a result, it demonstrates significant application potential in fields such as aero-engine thermal state monitoring, industrial temperature control, and complex multi-point temperature sensing. However, the practical implementation of multi-point array thin-film thermocouples faces several technical challenges [12,13]. Electromagnetic and thermal conduction coupling between adjacent sensing nodes can introduce signal interference, leading to measurement inaccuracies [14,15]. Furthermore, factors such as manufacturing consistency and the thermal contact behavior between sensing nodes and the substrate affect measurement reliability. These issues significantly hinder the engineering applicability of array thermocouples and require targeted solutions. In recent years, deep learning has been increasingly applied to address thermometric errors in thermocouples [16,17,18]. For example, Julian and James et al. [19] developed a deep wavelet neural network to model the nonlinear relationship between noise-filtered signals and actual temperatures, thereby improving measurement accuracy. Similarly, Payette, Julie et al. [20] proposed a deep learning framework for array-level temperature calibration to address inconsistencies and low precision in sensor arrays. Despite these advancements, calibration methods tailored to different temperature zones across multi-point array thin-film thermocouples remain underexplored and require further optimization.

To address the technical challenges outlined above, this study focuses on two key research directions: optimization of hardware fabrication and innovation in data calibration methods. At the hardware level, a precision welding process was used to fabricate the multi-point array thin-film thermocouple [21,22], and an insulating layer was applied to its surface via electrofluidic atomization. By optimizing welding parameters and process flows, the stability of the connections and the thermal response speed of the sensor nodes were improved. A multi-point synchronous temperature sensing hardware system was then developed, providing a solid foundation for accurate temperature field measurement. At the data processing level, to mitigate the impact of multi-sensor coupling on measurement accuracy, a deep learning-based temperature data calibration method was introduced. The results of this research are expected to facilitate the engineering deployment of multi-point array thin-film thermocouples in aerospace and industrial control applications. Additionally, this study offers valuable insights into the integration of temperature measurement technologies with artificial intelligence algorithms, demonstrating both theoretical significance and practical applicability.

## 2. Experimental

### 2.1. Materials

The thickness of the ceramic substrate is 1 mm. Ni-10%Cr wire (diameter 0.255 mm) and Ni-3%Si wire (outer diameter 0.255 mm) were produced by Yuguanghong Trading Co., Ltd. (Zhenzhou, China). The silicate composite material used to prepare the high-temperature insulating layer was purchased from Dongguan Huirui Electronics Co., Ltd. (Dongguan, China). The silicate composite material is mainly composed of CaO, Al_2_O_3_, and SiO_2_.

### 2.2. Equipments

Laser marking machine (OL-30w, Shandong Oulei Laser Technology Co., Ltd., Liaocheng, China). laser welding machine (AHL-W600III, Shenzhen Aohua Laser Technology Co., Ltd., Shenzhen, China) tube furnace (OTF-1200X, Hefei Kejing Materials Technology Co., Ltd., Hefei, China). 64-channel temperature data acquisition system (EX6000, Yili Technology Co., Ltd., Shenzhen, China). dynamic data acquisition and analysis system (DAQ6510, Tektronix, Inc., Beaverton, OR, USA). Scanning electron microscope (SUPRA55 SAPPHIRE, Carl Zeiss AG, Oberkochen, Germany).

### 2.3. Dataset

The main goal of this study is to design a general supervised calibration model $F:T_{rAw}\to T_{cAl}$. The model can effectively capture the nonlinear error mapping relationship between the raw measurement data from the array-type thermocouples and the true values of standard thermocouples, where $(T_{rAw}=t_{1},t_{2}\ldots t_{16})\in$ Input Feature Space represents the feature data input to the model (16 dimensions, including 10-dimensional time series features and 6-dimensional statistical features). To facilitate data collection, we conducted tests using a 32-point array thermocouple. In order to fully characterize the error patterns in the original measurement data through multidimensional features, we applied this model to 32 array-type thermocouple nodes arranged in a 4 × 8 matrix layout, achieving personalized error correction for thermocouple with different hardware characteristics.

**Figure 1:**
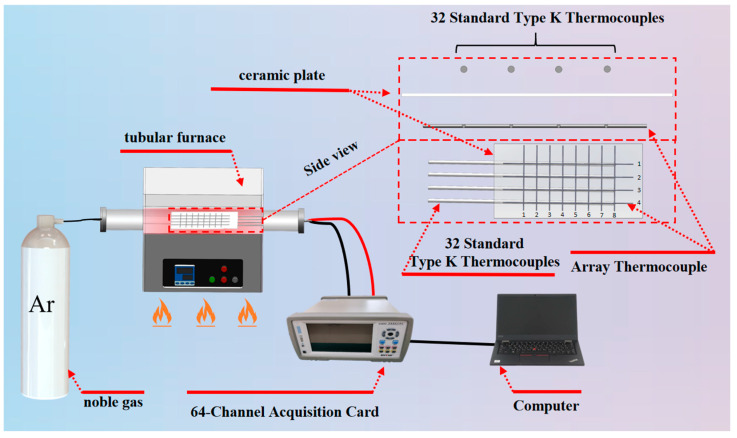
Schematic diagram of temperature data acquisition.

To obtain high-quality original temperature measurement data, this study constructed a three-level data collection system of “perception layer—collection layer—environment layer”. The schematic diagram of temperature data acquisition is shown in Figure 1. The equipment selection and function adaptation of each level are as follows:

① Perception Layer: Dual Thermocouple Array Deployment

The sensing layer comprises two types of thermocouple components. Due to the limited number of ports on the 64-channel acquisition card, a 4 × 8 multi-point array thermocouple configuration is adopted. One of these is a custom-fabricated 4 × 8 array thermocouple, which employs a nickel-chromium/nickel-silicon substrate and integrates 32 thermocouple nodes in a 4-row by 8-column matrix layout through a precision welding process. The node spacing is set to 7 mm to ensure uniform coverage of the temperature field inside the tubular high-temperature furnace, enabling synchronous acquisition of 32 channels of temperature measurement signals. The other component consists of 32 standard K-type thermocouples, which serve as the reference for true temperature values. Each standard thermocouple is aligned one-to-one with a corresponding node of the array thermocouple and secured using high-temperature-resistant adhesive. This arrangement ensures that the measuring junctions of both types of thermocouples are exposed to the same thermal environment, thereby eliminating measurement deviations caused by spatial position differences.

② Collection Layer: Multi-channel Synchronous Data Collection

For the acquisition layer, a 64-channel high-precision data acquisition card (model EX6000) was employed. The card is connected via USB to a host computer, enabling synchronous acquisition of 32 channels of array thermocouple signals and 32 channels of standard thermocouple signals. The sampling interval was set to 1 s to ensure temporal consistency in temperature readings and prevent timing deviations caused by acquisition delays.

③ Environment Layer: Temperature Control of Tube-type High-temperature Furnace

To simulate the high-temperature conditions typical of industrial environments, data acquisition was performed inside a tubular high-temperature furnace. For a detailed comparison of measurement accuracy across different thermal ranges, the experiments were divided into three distinct zones: low (0–400 °C), medium (400–700 °C), and high temperature (700–900 °C). A uniform heating rate of 8 °C/min was maintained throughout the tests. This rate reflects the actual heating processes commonly encountered in industrial equipment and ensures adequate thermal response time for the thermocouples, thereby avoiding measurement lag due to excessive temperature transients.

### 2.4. Performance Evaluation Methods for Deep Learning Models

To comprehensively and objectively evaluate the performance of the temperature calibration model for the 32-channel (4 × 8 layout) array thermocouple, a systematic and rigorous quantitative evaluation system was established. This system is designed to assess the model’s calibration accuracy, stability, and error-correction capability across multiple dimensions. Several core metrics with distinct evaluation focuses were selected to provide a multi-faceted characterization of model performance, thereby offering a scientific and quantifiable basis for validating its effectiveness.

Mean Absolute Error (MAE): The MAE serves as a core metric for evaluating the overall deviation between the calibrated temperature values and the standard reference values. It is computed as the average of the absolute differences across all samples, thereby avoiding the offsetting effect of positive and negative errors. This offers an intuitive reflection of the average accuracy of the model calibration. The formula is given as follows:

(1) $$MAE=\frac{1}{N}\sum_{i=1}^{N}\left|y_{i}-{\hat{y}}_{i}\right|$$

Among them, N is the number of samples, $y_{i}$ represents the true temperature value of the standard Type K thermocouple, ${\hat{y}}_{i}$ represents the calibrated temperature value output by the model. The smaller the MAE value, the better the overall calibration effect of the model.

Mean Squared Error (MSE): In contrast to MAE, the MSE places greater emphasis on penalizing larger deviations. By squaring the error terms, it amplifies the impact of significant outliers, thereby more effectively assessing the model’s capability to correct critical abnormal deviations and mitigating the influence of individual extreme errors on overall measurement accuracy. The calculation formula is as follows:

(2) $$\mathrm{MSE}=\frac{1}{N}{\sum_{i=1}^{N}\left(y_{i}-{\hat{y}}_{i}\right)}^{2}$$

Among them, N is the number of samples, $y_{i}$ represents the true temperature value of the standard Type K thermocouple, ${\hat{y}}_{i}$ represents the calibrated temperature value output by the model. The lower the MSE value, the better the model’s control effect on extreme deviations.

Coefficient of Determination (R^2^): The Coefficient of Determination (R^2^) is employed to quantify the goodness of fit between the calibrated temperature values and the standard reference values, reflecting the proportion of variance in the target variable that is predictable from the input features. It is calculated by comparing the sum of squared residuals to the total sum of squares, thereby evaluating the model’s ability to capture the underlying variation in the temperature data. The formula is expressed as follows:

(3) $$R^{2}=1-\frac{\sum_{i=1}^{N}{\left(y_{i}-{\hat{y}}_{i}\right)}^{2}}{\sum_{i=1}^{N}{\left(y_{i}-{\bar{y}}_{i}\right)}^{2}}$$

Among them, N is the number of samples, $y_{i}$ represents the true temperature value of the standard Type K thermocouple, ${\hat{y}}_{i}$ represents the calibrated temperature value output by the model, and ${\bar{y}}_{i}$ is the average value of the standard true values. The closer the R^2^ value is to 1, the higher the fitting degree between the calibrated data and the true value, and the more reliable the error correction law learned by the model.

Error Improvement Rate: The Error Improvement Rate serves as a direct indicator of the calibration method’s practical effectiveness. It quantifies the extent to which the model reduces the original measurement error by comparing the Mean Absolute Error (MAE) before and after calibration. The formula is defined as follows:

(4) $$\mathrm{Error}\;\mathrm{Improvement}\;\mathrm{Rate}=\frac{\mathrm{MAE}\;\mathrm{before}\;\mathrm{calibration}-\mathrm{MAE}\;\mathrm{after}\;\mathrm{calibration}}{\mathrm{MAE}\;\mathrm{before}\;\mathrm{calibration}}\times 100\%$$

Among them, MAE before calibration is the MAE between the original measured value of the array thermocouple and the standard true value, and MAE after calibration is the mean absolute deviation error between the calibrated temperature value and the measured value of the standard thermocouple used as the true value reference. The larger the improvement rate value, the more significant the effect of the calibration method on improving the measurement accuracy.

## 3. Results and Discussion

### 3.1. Preparation of Multi-Point Array Thin-Film Thermocouple

**Figure 2:**
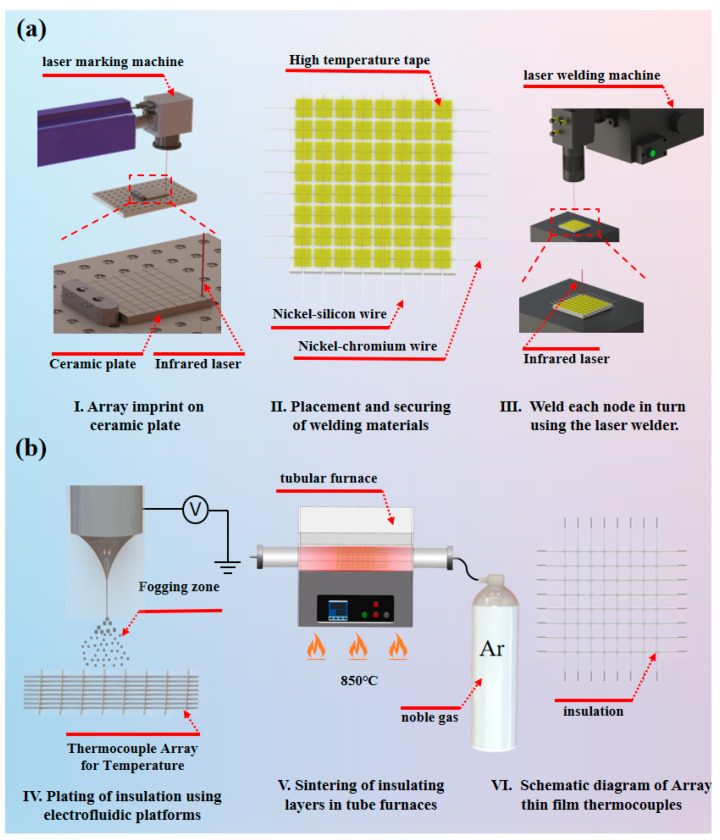
(**a**) Welding process of arrayed thermocouples. (**b**) Preparation of insulating layer on the surface of arrayed thermocouples.

**Figure 3:**
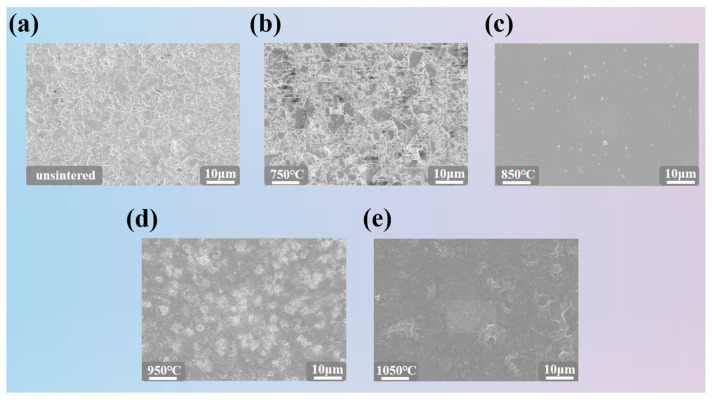
Close-up of microscopic morphology of arrayed thin film thermocouple surface insulation before sintering and after sintering at each temperature.

The multi-point array thin-film thermocouple features a simple structure composed of two elements: nickel-10% chromium wire and nickel-3% silicon wire. Figure 2a illustrates the welding process for the array thermocouple. First, laser marking machine etches the array’s conductor traces onto the ceramic substrate, ensuring consistent spacing between materials during subsequent placement of welding materials. Subsequently, the nickel-chromium wire and nickel-silicon wire are sequentially placed onto the marked 8 × 8 array conductor traces and secured with high-temperature tape to guarantee uniform wire spacing. Finally, the secured NiCr and NiSi wires are positioned under the laser welding machine. After multiple welding parameter tests, the final settings are as follows: Spot size controlled to 0.2 mm via the modulation knob, voltage set to 75 V, pulse width at 2.5 ms, the continuous emission count is one time, and continuous emission frequency at 12 Hz [23,24]. This parameter set enables stable welding of the junctions. Simultaneously, the inert shielding gas (argon) channel was activated and aligned with the weld point. This prevented excessive thermal stress during heating from causing oxidation and embrittlement of the sensitive electrodes, ensuring overall welding stability and precision. Subsequently, each junction was welded sequentially [25,26], followed by final quality and connection reliability testing. Figure 2b shows the preparation process of the insulating layer on the surface of the array thin-film thermocouple [27,28]. First, the atomization mode of the self-built electrohydrodynamic inkjet platform is used to coat the insulating layer on the array thermocouple [29,30], where the insulating layer is composed of silicate composite ceramics and a diluent at a ratio of 10:1 [31,32,33]. Then, after the coated array thermocouple is cured at room temperature, it is placed in a tube furnace for high-temperature sintering [34], finally forming a dense, stable solid structure with excellent insulating performance and mechanical strength for the insulating layer. For performance testing of array thermocouples, please refer to the Supplementary Information. To ensure that the insulating layer on the surface of the array thin-film thermocouple achieves stable and high-strength effects, high-temperature sintering of the insulating layer is required. As the temperature increases, the inorganic particles in the insulating layer slurry begin to sinter, and diffusion and bonding occur between the particles to form a dense solid structure. In this stage, the pores between particles gradually decrease, and the mechanical strength and electrical insulation performance of the material are significantly improved. Figure 3 shows the microscopic morphology close-ups of the insulating layer on the surface of the array thin-film thermocouple sintered at 750 °C, 850 °C, 950 °C, and 1050 °C. As shown in Figure 3b,d,e, the insulating material is highly sensitive to the sintering temperature. The optimal sintering temperature was determined according to the following criteria: (i) continuous and defect-free surface coverage (minimal cracking/porosity) observed in SEM, and (ii) stable electrical insulation performance (high insulation resistance/low leakage) after sintering. Insufficient sintering may result in incomplete grain growth, whereas excessive temperature can cause excessive particle diffusion and local surface damage, leading to larger voids or discontinuities. In addition, the specimen must be sintered under a uniform temperature field; otherwise, the insulating layer may crack, delaminate, or degrade, resulting in failure. Based on experimental optimization (Figure 3c), the optimal sintering condition was determined to be 850 °C with a heating/cooling rate of 5 °C/min and a holding time of 20 min. During sintering, organic components (e.g., binders) decompose and volatilize first, leaving a purified inorganic phase. With further temperature increase, inorganic particles undergo diffusion and bonding, forming a dense solid structure with gradually reduced porosity, which significantly improves the mechanical strength and electrical insulation performance. Near the sintering temperature, phase transformation occurs, producing a more stable crystalline structure. Grain growth continues in this stage, leading to a uniform microstructure. Such phase evolution typically involves a transition from an amorphous or poorly ordered state to a more ordered crystalline phase, thereby enhancing the thermal stability and dielectric properties of the insulating material.

### 3.2. Deep Learning Models

**Figure 4:**
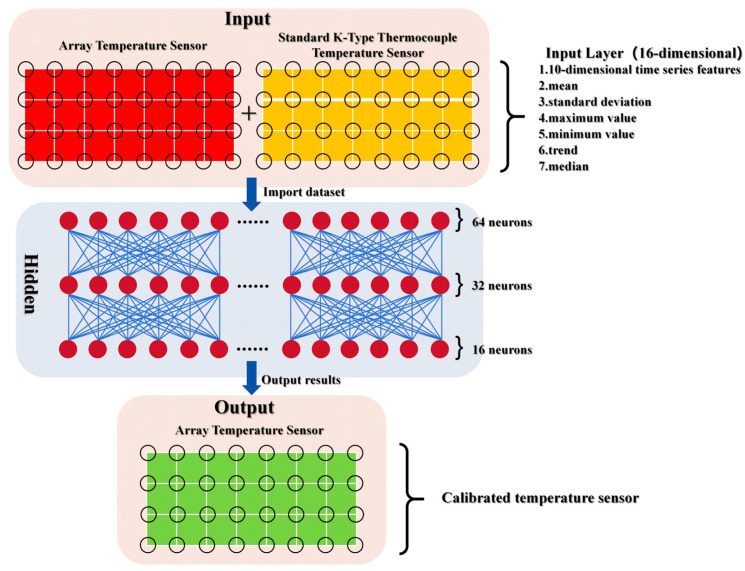
Schematic diagram of array thermocouple temperature calibration model architecture based on MLP.

To address the measurement deviations in the 32 self-fabricated array thermocouples caused by multi-node coupling effects and variations in hardware characteristics, this study designs a deep learning calibration method based on a Multi-Layer Perceptron (MLP). The model follows the classic fully connected architecture of “input layer—hidden layer—output layer”. A schematic diagram of the MLP-based array thermocouple temperature calibration model is shown in Figure 4. In this method, the readings from the standard thermocouple are taken as the reference true values. An input space integrating time-series features and statistical characteristics is constructed, and an individual calibration model is trained for each self-made sensor node. This approach enables accurate correction of measurement errors, with the core objective of achieving an average error reduction rate of 30–70% while maintaining the coefficient of determination (R^2^) stably above 0.8.

During the data preprocessing phase, the original data were obtained from synchronous acquisitions by a tubular high-temperature furnace, involving both the array thermocouple (Channels 1–32) and a standard K-type thermocouple (Channels 33–64). A standardized three-step procedure—comprising format verification, outlier removal, and feature engineering—was systematically applied. For format verification, an encoding-adaptive mechanism was employed to resolve data reading errors caused by mixed encoding formats. Specifically, decodings were attempted in sequence using UTF-8, GBK, GB2312, and Latin-1. Based on the channel correspondence (Channel 1 ↔ Channel 33, …, Channel 32 ↔ Channel 64), the readings from each array thermocouple and their matched standard thermocouple were paired into sample sets, ultimately forming 32 independent datasets. In outlier elimination, the interquartile range (IQR) method was applied to each dataset. The first quartile (Q1) and third quartile (Q3) were calculated, and the interval [Q1 − 1.5 × IQR,Q3 + 1.5 × IQR] (where IQR = Q3 − Q1) was defined as the valid data range. Any extreme values lying outside this range were removed to prevent interference with model training. During feature engineering, a 16-dimensional input feature vector was constructed. This included 10 time-series features, formed by extracting the raw measurements from the past 10 consecutive time points via a sliding window, thereby capturing the dynamic trend of temperature variation. Additionally, 6 statistical features—mean, standard deviation, maximum, minimum, trend value, and median—were derived from the time-series data to enrich the feature representation and support robust model learning. Among these, the overall ablation test compares the reference support material.

During the model training phase, in light of the heterogeneous error characteristics among the 32 thermocouple channels—resulting from variations in welding quality, thermal contact performance, and thermal response speed—a coordinated training strategy of “individual training + unified protocol” was adopted. For each sensor, the preprocessed dataset was split into a training set and a validation set at an 8:2 ratio. The training set was used to learn the nonlinear mapping between the raw measurement values and the standard reference values, while the validation set served to monitor potential overfitting and prevent evaluation bias caused by data leakage. In terms of training configuration, the Adam optimizer was employed, which dynamically adjusts the learning rate using first- and second-moment estimates of the gradient to enhance training stability. The initial learning rate was set to 0.001 to balance convergence speed and update precision. Training was conducted over a maximum of 500 epochs, with an early stopping strategy applied (patience = 20), halting the process if the validation loss failed to improve for 20 consecutive epochs to avoid overfitting. The mean squared error (MSE) was used as the loss function, which strongly penalizes large prediction errors, thereby guiding the model to focus on mitigating significant deviations. An independent MLP model was trained for each sensor, with parameters individually initialized and updated. This approach ensures that each model accurately adapts to the specific error behavior of its corresponding sensor, collectively enhancing the overall calibration performance across all 32 thermocouple channels.

### 3.3. Model Calibration Results

**Figure 5:**
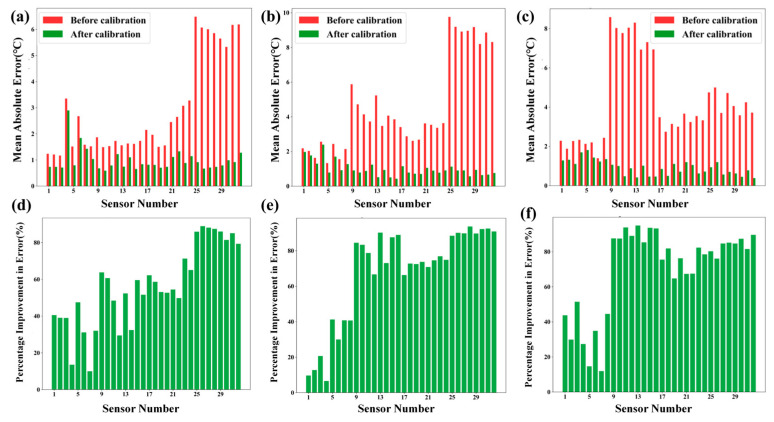
(**a**) Low-temperature zone error distribution diagram. (**b**) Mid-temperature zone error distribution diagram. (**c**) High-temperature zone error distribution diagram. (**d**) Low-temperature zone accuracy enhancement distribution diagram. (**e**) Mid-temperature zone accuracy enhancement distribution diagram. (**f**) High-temperature zone accuracy enhancement distribution diagram.

Figure 5 presents the error distribution before and after calibration, along with the corresponding percentage improvement in error for the array thermocouples across three temperature ranges: low, medium, and high. Figure 5a–c show the Mean Absolute Error (MAE) before and after calibration. The red bars represent the MAE before calibration, which remain relatively high and exhibit a scattered distribution across all sensor nodes. After calibration, as indicated by the green bars, the MAE is significantly reduced, with most errors falling below 2 °C and centered around 1 °C in all temperature ranges. Specifically, in the low-temperature range, errors before calibration approach 6 °C, while post-calibration errors drop to below 2 °C. In the medium-temperature range, errors initially reach up to 8 °C, but after calibration, they are also reduced to under 2 °C. In the high-temperature range, most errors are between 3 and 5 °C before calibration, but again, the errors are substantially reduced post-calibration. Figure 5d–f show the percentage improvement in error after calibration. The green bars represent the improvement in error for each sensor in the low-, medium-, and high-temperature ranges. In the low-temperature range, most sensors exhibit error improvements between 40% and 80%. In the medium-temperature range, nearly all sensors show an improvement of over 60%, with a significant portion reaching nearly 80%. In the high-temperature range, the improvement ratios are mainly between 40% and 80%, with a more uniform distribution. Overall, the calibration method proves highly effective across the entire temperature spectrum, with the medium-temperature range showing the most pronounced improvement due to initially higher errors, while the high-temperature range demonstrates the best uniformity in post-calibration error and improvement ratio due to more evenly distributed original errors.

**Figure 6:**
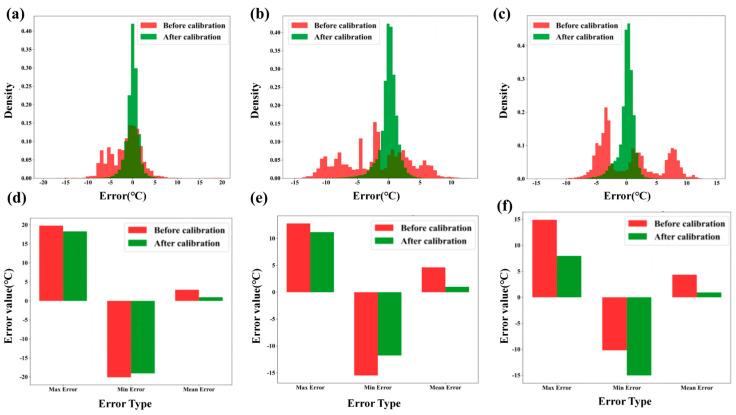
(**a**) Low-temperature zone error distribution histogram. (**b**) Mid-temperature zone error distribution histogram. (**c**) High-temperature zone error distribution histogram. (**d**) Low-temperature zone overall error statistics comparison. (**e**) Mid-temperature zone overall error statistics comparison. (**f**) High-temperature zone overall error statistics comparison.

Figure 6 presents data regarding the distribution of errors before and after calibration, along with corresponding error value changes for maximum, minimum, and mean errors across different temperature ranges. Figure 6a–c: These panels display histograms of the error distribution before and after calibration for the low, medium, and high-temperature ranges, respectively. The red bars represent the error density before calibration, while the green bars represent the error density after calibration. In each plot, it is evident that the error distribution is highly skewed to higher error values before calibration, with significant peaks around 0 °C in the post-calibration data. Calibration results in a noticeable shift in the error distribution towards smaller values, reducing the overall spread of the errors across the temperature ranges, indicating improved measurement accuracy. Figure 6d–f: These panels show bar graphs comparing the maximum, minimum, and mean error values before and after calibration for the low, medium, and high-temperature ranges. In all temperature ranges, the maximum error (red bars) is significantly reduced after calibration (green bars). The minimum error also improves, with post-calibration values being closer to zero. The mean error decreases considerably, demonstrating the overall improvement in measurement accuracy after calibration across the entire temperature spectrum. Overall, the data clearly demonstrate that the calibration process leads to substantial reductions in both error magnitude and variability, improving the accuracy and consistency of the thermocouple measurements across all temperature ranges.

**Figure 7:**
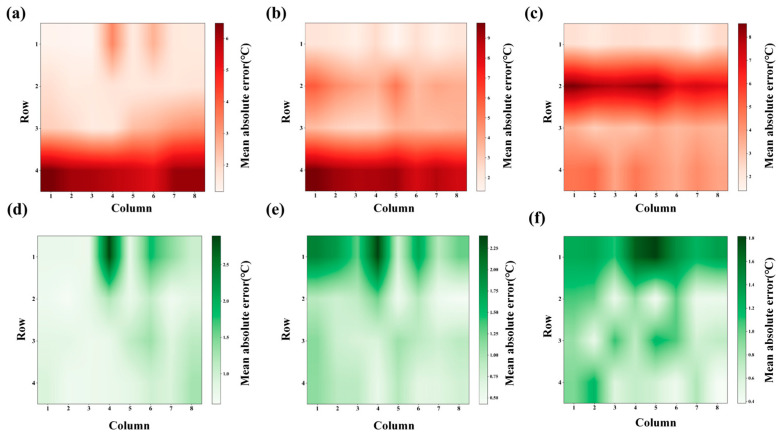
(**a**) Low-temperature zone distribution of errors before calibration. (**b**) Mid-temperature zone distribution of errors before calibration. (**c**) High-temperature zone distribution of errors before calibration. (**d**) Low-temperature zone distribution of errors after calibration. (**e**) Mid-temperature zone distribution of errors after calibration. (**f**) High-temperature zone distribution of errors after calibration.

Figure 7 presents heatmaps illustrating the Mean Absolute Error (MAE) distribution across the sensor array before and after calibration in a grid format, with rows and columns representing different sensor positions. Figure 7a–c: These panels show the MAE distribution before calibration for the low, medium, and high-temperature ranges, respectively. The red color indicates higher MAE values, with areas of the sensor grid showing significant error concentrations. In the low- and medium-temperature ranges (a and b), the error is most pronounced in certain sensor positions, especially in the upper rows of the grid, with the maximum MAE reaching above 5 °C. In the high-temperature range (c), the error distribution is more uniform, with values generally ranging between 2 and 7 °C, although certain areas still show higher errors. Figure 7d–f: These Figures show the MAE distribution after calibration for the low, medium, and high-temperature ranges. After calibration, the error values (shown in green) are significantly reduced across the entire sensor array. The MAE now remains below 3 °C for the low-temperature range (d), below 2 °C for the medium-temperature range (e), and below 2 °C for the high-temperature range (f), with the errors more evenly distributed across the grid. The calibration process has notably improved the measurement accuracy, reducing localized high errors and enhancing the overall consistency of the sensor performance. These heatmaps demonstrate the effectiveness of the calibration process in reducing the MAE and improving the uniformity of the thermocouple array’s performance across various temperature ranges. The most significant improvements are observed in the high-error regions, which were concentrated in specific sensor areas before calibration.

**Figure 8:**
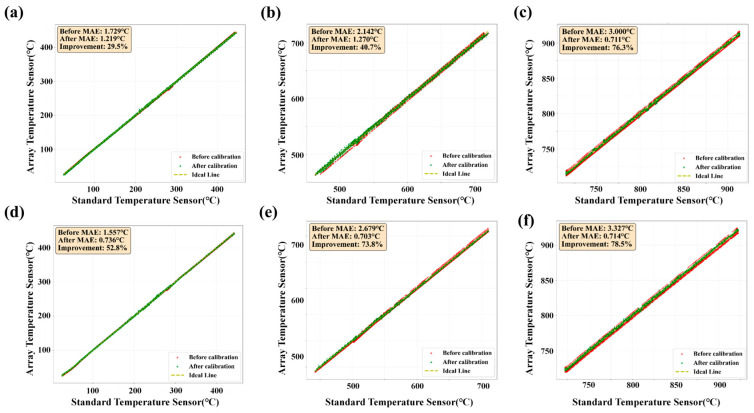
(**a**) Single-node performance enhancement curve for low-temperature zones in array thermocouples. (**b**) Single-node performance enhancement curve for mid-temperature zones in array thermocouples. (**c**) Single-node performance enhancement curve for high-temperature zones in array thermocouples.

Figure 8 compares the temperature measurement performance of the array sensor with that of a standard reference sensor in the low-, medium-, and high-temperature regions, where calibration results from two randomly selected nodes in each region are shown. In the low-temperature region (Figure 8a,d), the uncalibrated readings deviate visibly from the ideal line (perfect agreement with the reference), yielding an MAE of 1.729 °C; after calibration, the data cluster much closer to the ideal line and the MAE decreases to 1.219 °C, corresponding to a 29.5% improvement. In the medium-temperature region (Figure 8b,e), the pre-calibration deviation is more pronounced, with an initial MAE of 2.142 °C, whereas calibration substantially improves the agreement and reduces the MAE to 1.270 °C, achieving a 40.7% improvement. In the high-temperature region (Figure 8c,f), the largest discrepancy is observed before calibration (MAE = 3.000 °C); after calibration, the measurements align closely with the reference, reducing the MAE to 0.711 °C and delivering a 76.3% improvement. Overall, calibration consistently brings the array sensor outputs closer to the reference values across all temperature ranges, with the greatest relative gain occurring at high temperatures, indicating that the proposed method not only improves accuracy over the full range but also becomes increasingly effective as temperature increases. In addition, we have provided the MAE values, error improvement rates and R^2^ values before and after calibration over the entire temperature range in Table 1.

In summary, the deep learning-based calibration method using a 3-hidden-layer MLP model demonstrates excellent performance in enhancing the measurement accuracy of multi-point array thin-film thermocouples. Through systematic training and evaluation across low-, medium-, and high-temperature zones, the proposed approach effectively compensates for measurement deviations caused by multi-node coupling effects and variations in hardware characteristics. After calibration, the mean absolute error (MAE) of all 32 sensor nodes is significantly reduced to below 2 °C, with an average error improvement rate ranging from 30% to 80%. The coefficient of determination (R^2^) remains stable between 0.8 and 0.95, reflecting a strong fit between the calibrated outputs and the standard reference values. In terms of data characteristics, the error distribution of the sensor nodes shifts from widely dispersed to tightly concentrated at low values. The most substantial accuracy improvement is observed in the high-temperature zone, which also exhibits the most uniform error reduction effect. Notably, even for individual nodes with severe initial deviations, the model achieves targeted correction through tailored training. These results collectively validate the reliability, stability, and engineering applicability of the proposed calibration method, providing a solid experimental foundation for the practical deployment of multi-point array thin-film thermocouples in high-precision temperature field monitoring scenarios.

## 4. Conclusions

This study developed and implemented an MLP-based calibration system for self-fabricated thermocouples, enabling high-precision calibration of multi-point array thin-film thermocouples. A three-stage data preprocessing pipeline—format verification, outlier removal, and feature engineering—was established to improve data quality and training robustness. In particular, an encoding-adaptive mechanism effectively addressed data parsing anomalies caused by heterogeneous file encodings, while an IQR-based strategy filtered extreme temperature values to reduce noise interference. To comprehensively characterize the temperature signals, a 16-dimensional feature vector was constructed to capture both dynamic trends and statistical attributes, providing informative inputs for model learning. An independent MLP with an input–hidden–output architecture was trained using the Adam optimizer (initial learning rate 0.001) with a maximum of 500 iterations and an early-stopping criterion (patience = 20) on an 8:2 training–validation split. Experimental results show that the proposed method achieves an average error reduction of 30–70% across the self-fabricated thermocouples, maintains stable predictive performance with R^2^ values between 0.80 and 0.95, and significantly suppresses maximum error. In addition, the system automatically generates analytical plots and detailed calibration reports to visualize and document calibration outcomes. Overall, the results demonstrate that the proposed deep learning–based calibration system provides an accurate and efficient solution for calibrating self-fabricated thermocouples, offering a reliable approach to improving temperature measurement accuracy in practical applications.

## Figures and Tables

**Table 1:** Detailed Error Information for Each Node.

Node Number	MAE Before Calibration	MAE After Calibration	Error Improvement Rate	R^2^
Node 1	2.3835	1.6665	30.1	0.99
Node 2	2.8569	2.3641	17.2	0.99
Node 3	2.4241	1.6823	30.6	0.99
Node 4	3.2862	2.3339	29.0	0.99
Node 5	2.3137	1.3985	39.6	0.99
Node 6	3.0599	1.9844	35.1	0.99
Node 7	2.3117	1.2749	44.9	0.99
Node 8	2.6545	1.4402	45.7	0.99
Node 9	4.5173	1.2002	73.4	0.99
Node 10	4.0784	1.1072	72.9	0.99
Node 11	3.9317	1.0595	73.1	0.99
Node 12	4.0697	1.2299	69.8	0.99
Node 13	4.3193	0.8613	80.1	0.99
Node 14	3.7761	1.2886	65.9	0.99
Node 15	3.9219	0.8606	78.1	0.99
Node 16	3.7730	0.9953	73.6	0.99
Node 17	4.7545	1.5618	67.2	0.99
Node 18	4.2583	1.3976	67.2	0.99
Node 19	4.0953	1.4270	65.2	0.99
Node 20	4.1793	1.5410	63.1	0.99
Node 21	4.9275	2.0494	58.4	0.99
Node 22	4.6707	1.8226	61.0	0.99
Node 23	4.6944	1.2083	74.3	0.99
Node 24	4.6399	1.1418	75.4	0.99
Node 25	8.5431	1.7356	79.7	0.99
Node 26	8.2296	1.5270	81.4	0.99
Node 27	7.8452	1.4285	81.8	0.99
Node 28	8.2161	1.3286	83.8	0.99
Node 29	7.6585	1.6266	78.8	0.99
Node 30	7.4237	1.5904	78.6	0.99
Node 31	7.8808	1.1657	85.2	0.99
Node 32	7.7437	1.0898	85.9	0.99

## Data Availability

The original contributions presented in this study are included in the article. Further inquiries can be directed to the author.

## Supplementary Materials

The following supporting information can be downloaded at: https://www.mdpi.com/article/10.3390/s26061956/s1, Figure S1: Schematic Diagram of Array Thermocouple Dimensions; Figure S2: Physical image of the array thermocouple; Figure S3: Schematic diagram of self-built electrohydraulic flow platform; Figure S4: Array thermocouple test platform; Figure S5: Schematic diagram of the response time test platform; Figure S6: Schematic diagram of high-temperature flame gun thermal shock test bench; Figure S7: Hysteresis of the array thermocouple; Figure S8: Accuracy of the array thermocouple; Figure S9: Fitting error of the array thermocouple; Figure S10: Drift rate of the array thermocouple; Figure S11: Fitting curve of the array thermocouple; Figure S12: Result of response time in the array thermocouple; Figure S13: Result of cycle test in the array thermocouple; Figure S14: Result of high temperature flame in the array thermocouple; Figure S15: Overall Ablation Comparison; Table S1: Deep Learning Model Architecture.

## Abbreviations

The following abbreviations are used in this manuscript:

MLP	Multi-Layer Perceptron
MAE	Mean Absolute Error
MSE	Mean Squared Error
R^2^	Coefficient of Determination
