# Supplementary material for: Deep Learning-Based Calibration of a Multi-Point Thin-Film Thermocouple Array for Temperature Field Measurement
Source: Sensors (Basel). 2026 Mar 20;26(6):1956. doi: 10.3390/s26061956 (PMC13030179; doi:10.3390/s26061956)
Supplement: Supplementary file 1 [file sensors-26-01956-s001.zip › sensors-4199680-supplementary.pdf]

Supplementary Information

Deep Learning-Based Calibration of a Multi-Point Thin-Film Thermocouple Array for Temperature Field Measurement

Zewang Zhang [1,*], Shigui Gong [1], Jiajie Ye [1], Chengfei Zhang [2], Jun Chen [3], Zhixuan Su [4], Heng Wang [4], Zhichun Liu [4,*] and Zhenyin Hai [4,*]

[1] School of Opto-Electronic and Communication Engineering, Xiamen University of Technology, Xiamen 361005, China
[2] Inner Mongolia Aerospace Power Machinery Testing Institute, Hohhot 010076, China
[3] School of Electronic Engineering, Ocean University of China, Qingdao 266100, China
[4] School of Aerospace Engineering, Xiamen University, Xiamen 361005, China
* Correspondence: zwzhang@xmut.edu.cn (Z.Z.); liuzhichun@xmu.edu.cn (Z.L.); haizhenyin@xmu.edu.cn (Z.H.)

Supplementary Note 1: Performance test method

The primary performance parameters of the thermocouple are hysteresis, stability/drift, accuracy, and response time. Hysteresis refers to the offset between the forward temperature excitation and the reverse temperature excitation at the thermocouple output value at a given temperature. Hysteresis is calculated by the following formula:

$$\text{Hysteresis}=\frac{\Delta T|_{V=x}}{T_{F.S.}}\times 100\%$$

Where $\Delta T|_{V=x}$ denotes the deviation ΔT between the forward and reverse temperature excitations at the thermocouple output voltage value, and $T_{F.S.}$. denotes the calibration temperature range.

Stability/drift represents the ability of the thermocouple to maintain its performance parameters over a certain period of time. Stability/drift is calculated as follows:

$$\text{Drift/Stability}=\frac{\Delta T_{CT}|_{Temp=x,Time=y}-\Delta T_{B}|_{Temp=x,Time=y}}{T_{F.S}}\times 100\%$$

Where $\Delta T_{CT}|_{Temp=x,Time=y}$ denotes the change in output temperature value of array thin-film thermocouples at a temperature of x °C and a holding time of y hours. $\Delta T_{B}|_{Temp=x,Time=y}$ denotes the change in output temperature value of a standard type B thermocouple at a temperature of x °C and a holding time of y hours. $T_{F.S}$ denotes the full-scale temperature.

Accuracy is calculated by the following formula:

$$\text{Accuracy}=\frac{\Delta T}{T_{F.S.}}\times 100\%$$

Where ΔT denotes the temperature deviation between the thermocouple being measured and the standard thermocouple, and $T_{F.S.}$ denotes the calibration temperature range.

The response time ($\tau_{0.632}$) is calculated using the following formula:

$$\tau_{0.632}=\Delta t\times 63.2\%$$

Where Δt denotes the time for the thermocouple to reach a steady state after being excited by a heat source.

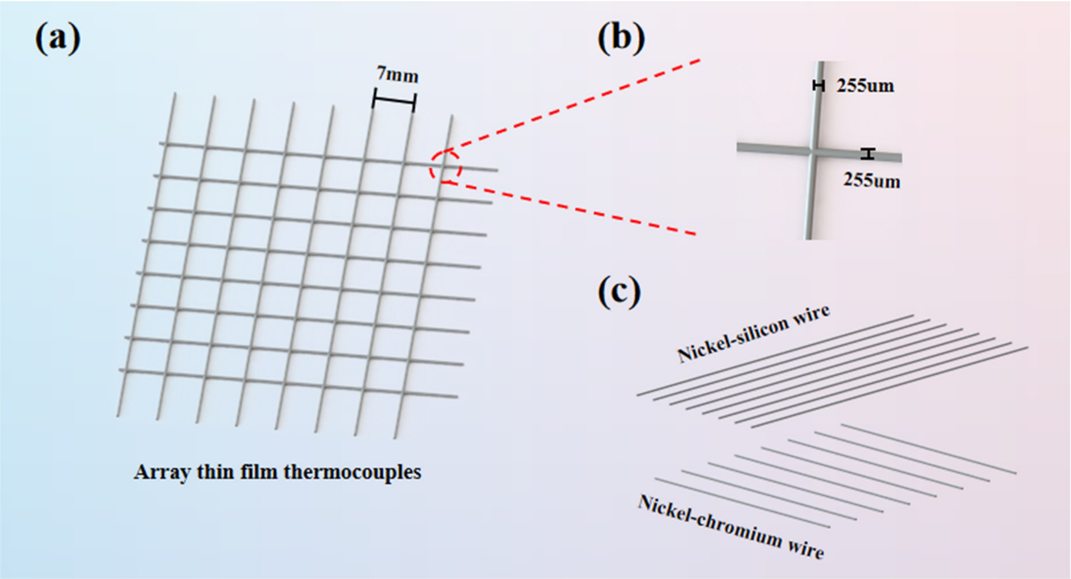

Figure S1.(a) Spacing between arrayed thin-film thermocouple wires; (b) diameters of NiCr and NiSi wires; (c) exploded view of arrayed thin-film thermocouple.

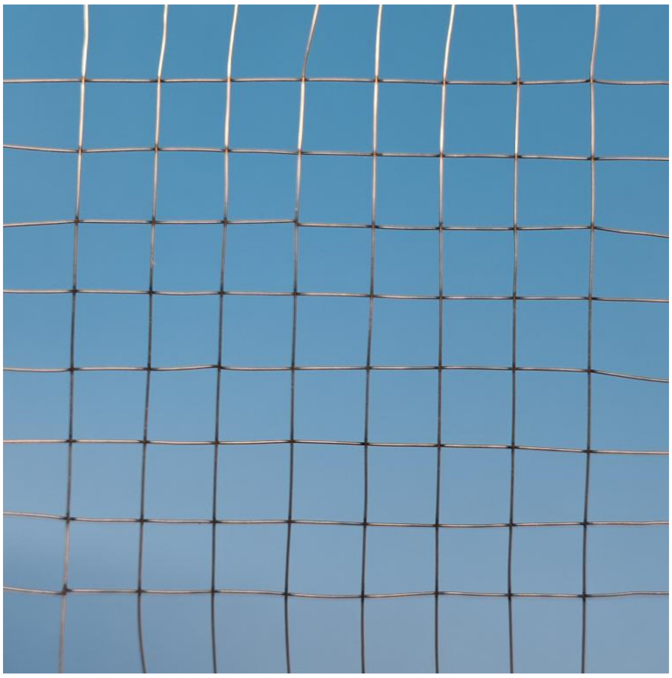

Figure S2. Physical image of the array thermocouple.

Supplementary Note 2: Self-built electrofluidic platform

As shown in **Figure S3**, The self-built electrohydrodynamic inkjet platform mainly consists of a programmable three-dimensional motion platform, a micro-injection pump, a metal needle, a high-voltage power supply, a computer, and a microscopic imaging acquisition module. The three-dimensional motion platform can achieve precise positioning of the substrate. The metal needle is fixed on the injection

pump and connected to the high-voltage power supply, which can supply ink to the needle tip. Under the combined action of electric field force, surface tension, viscous force, and liquid hydrostatic pressure, a stable Taylor cone and nano-scale ultra-fine spray can be formed. Adjusting the angle of the camera can capture the conical jet on the needle tip, which finally deposits on the substrate to form a continuous film.

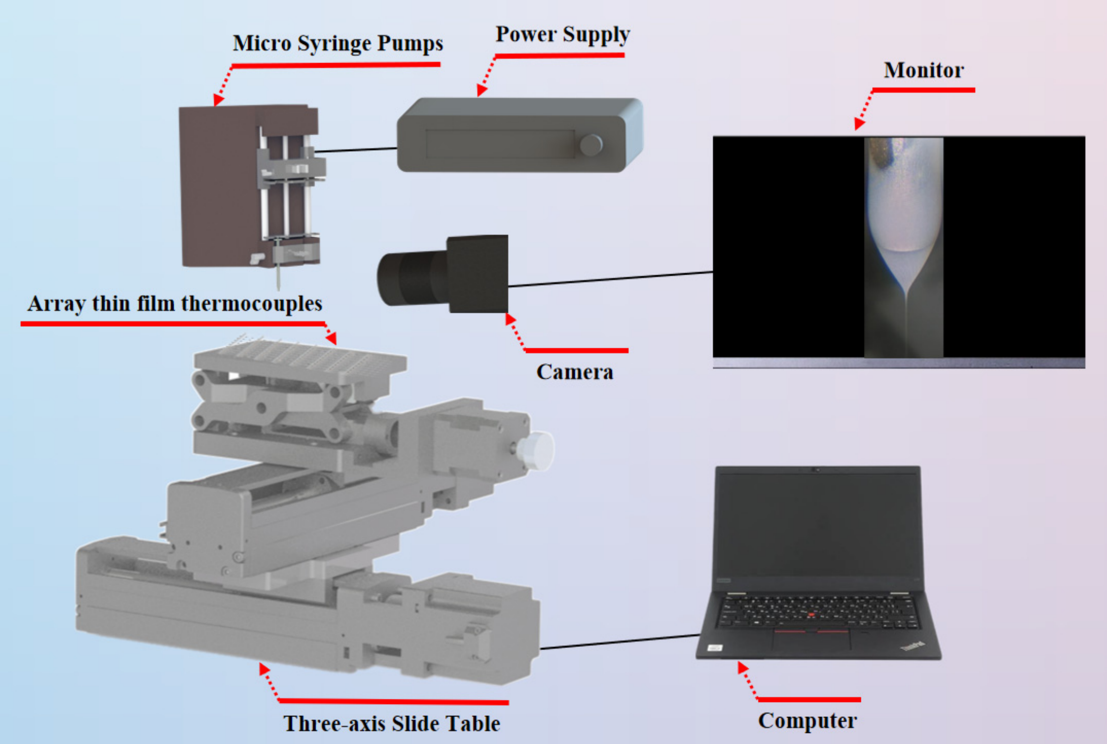

Figure S3. Schematic diagram of self-built electrohydraulic flow platform.

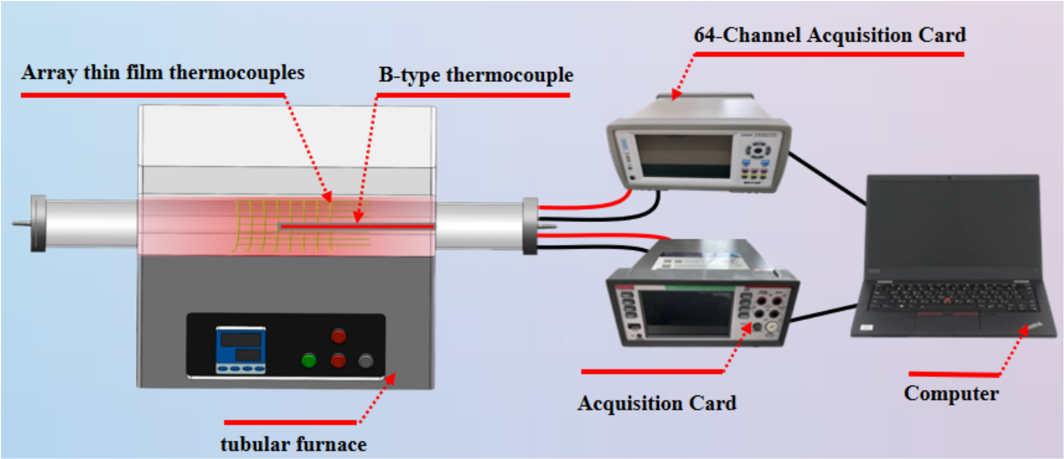

Figure S4. Array thermocouple test platform.

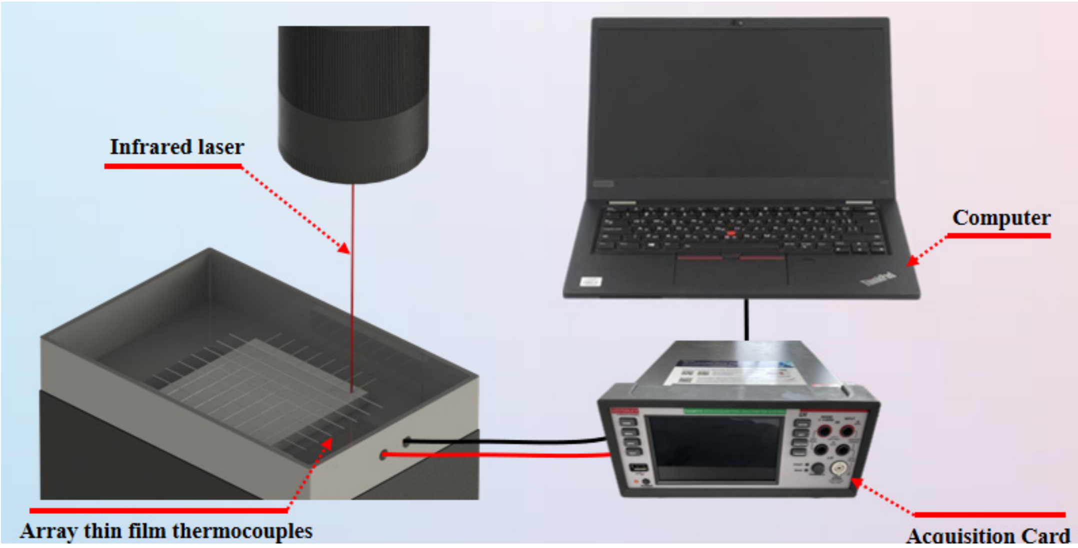

Figure S5. Schematic diagram of the response time test platform.

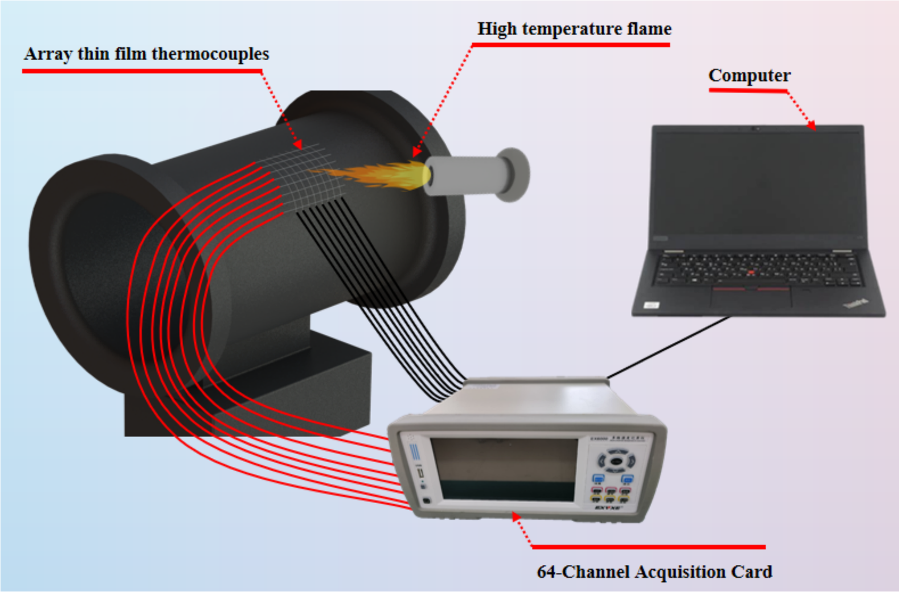

Figure S6. Schematic diagram of high-temperature flame gun thermal shock test bench.

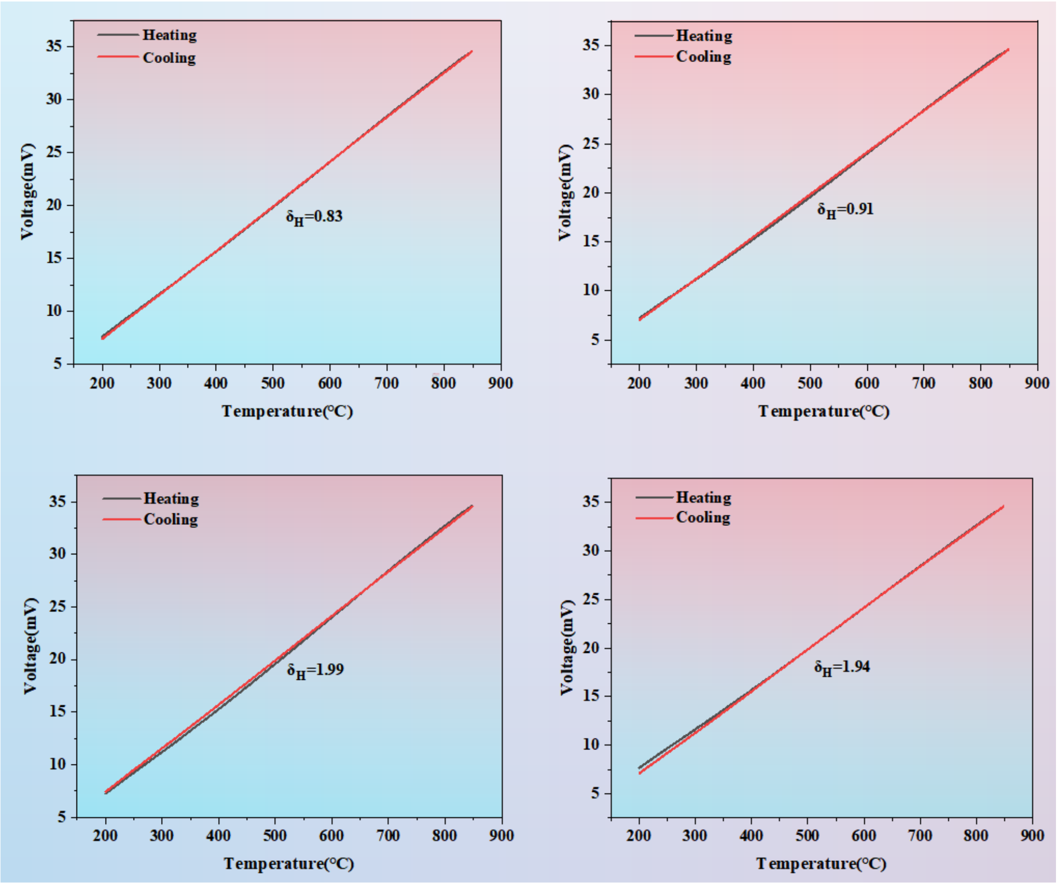

Figure S7. Hysteresis of the array thermocouple.

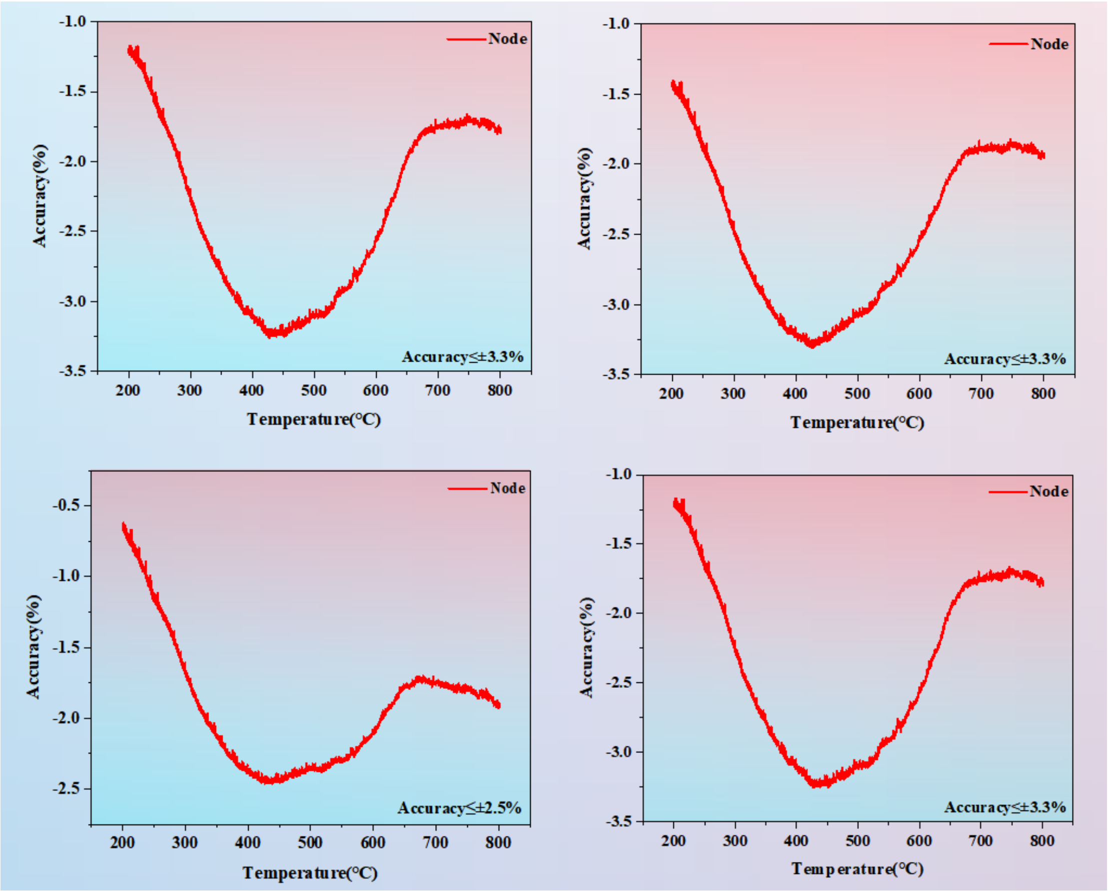

Figure S8. Accuracy of the array thermocouple.

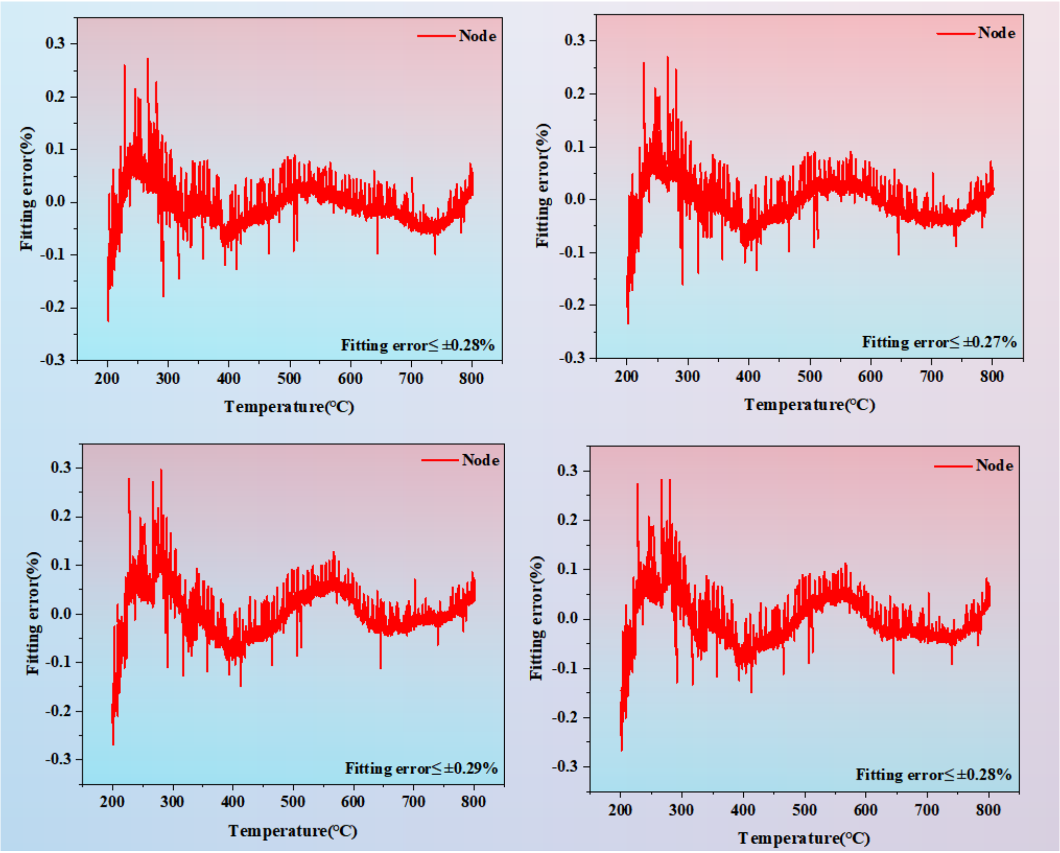

Figure S9. Fitting error of the array thermocouple.

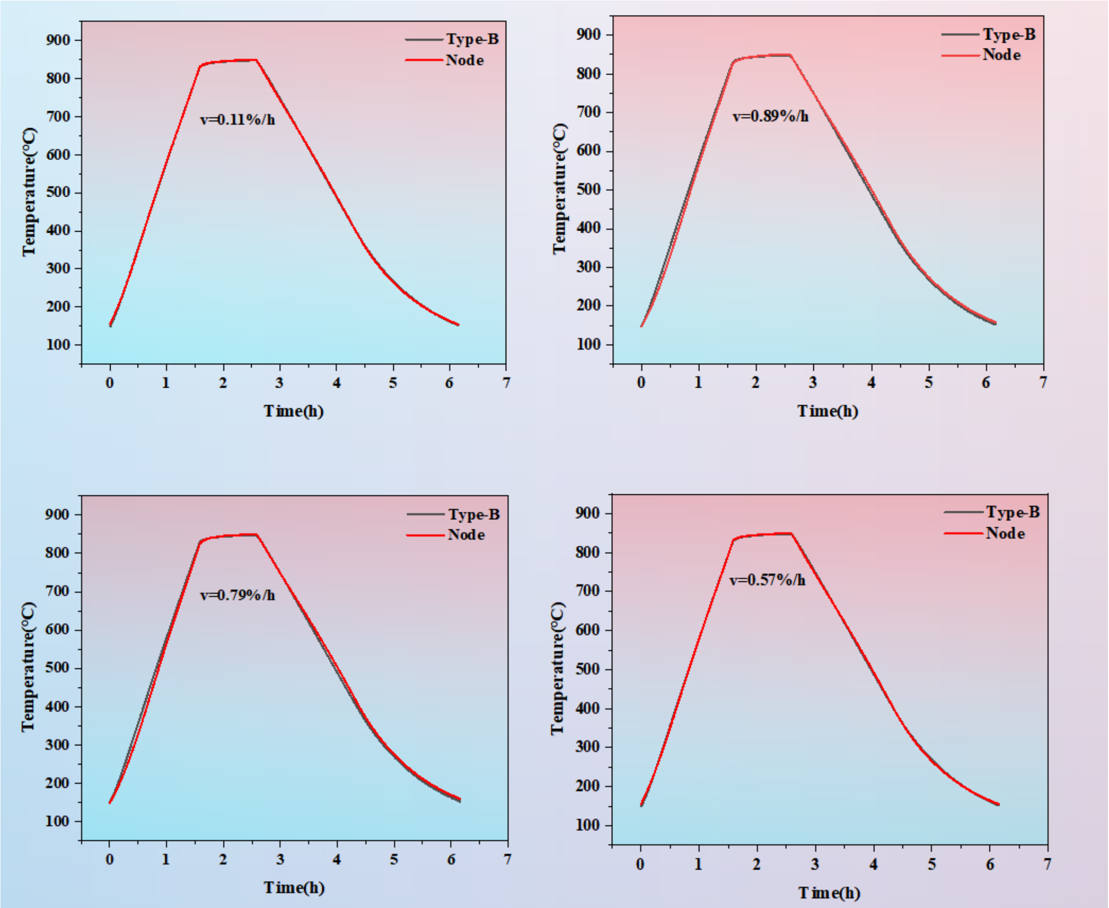

Figure S10. Drift rate of the array thermocouple.

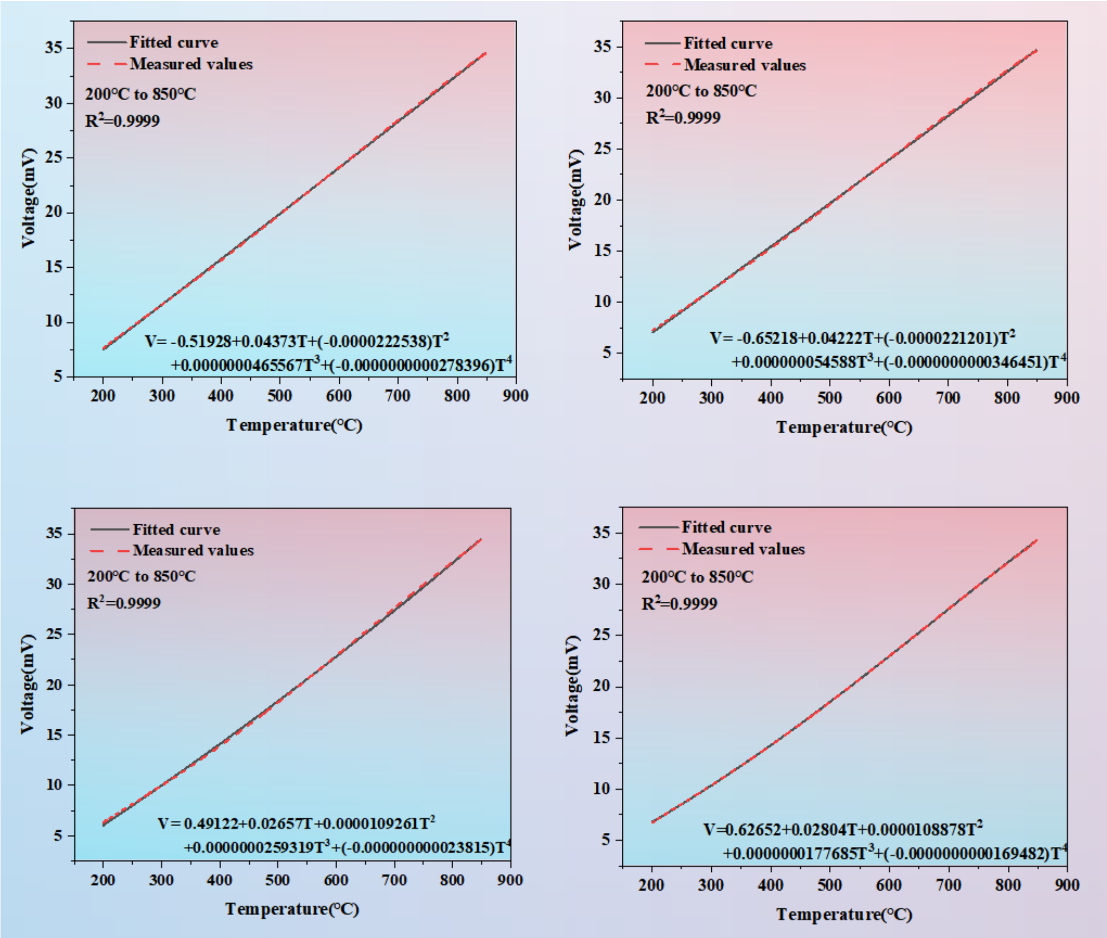

Figure S11. Fitting curve of the array thermocouple.

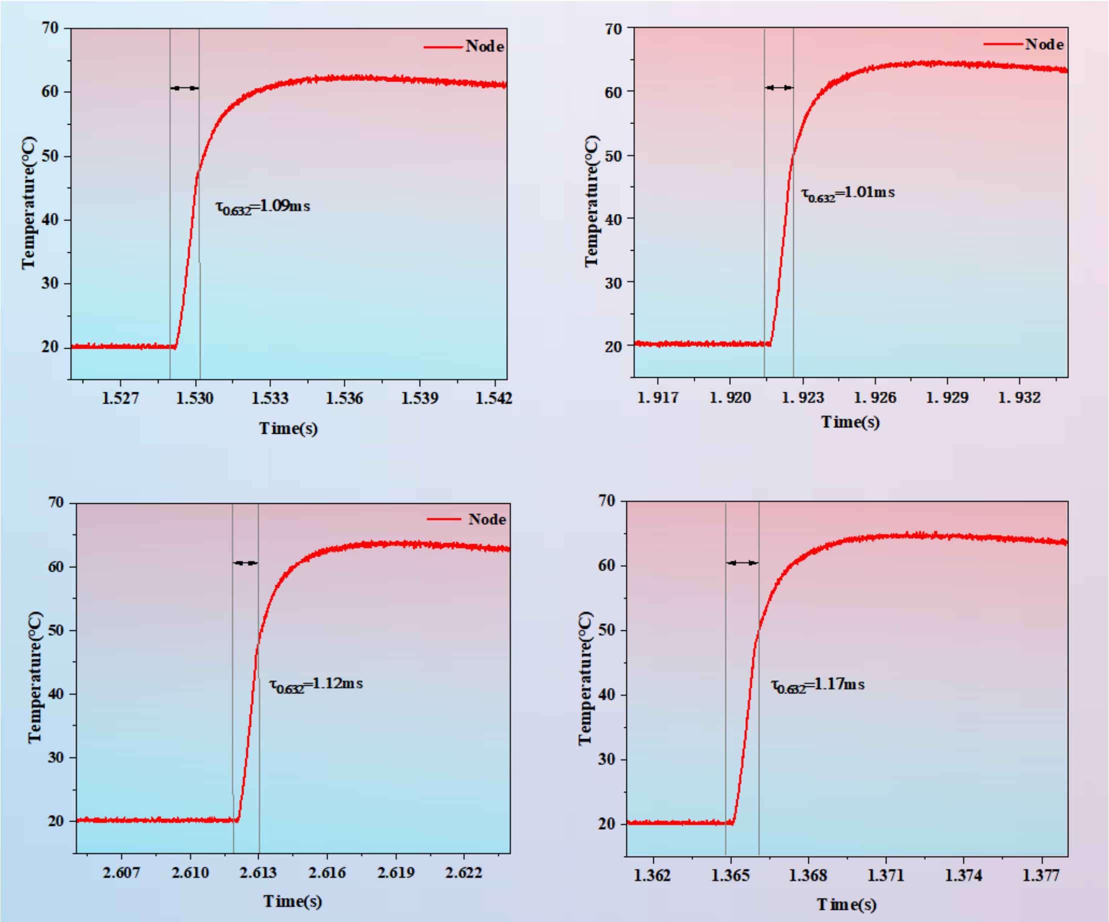

Figure S12. Result of response time in the array thermocouple.

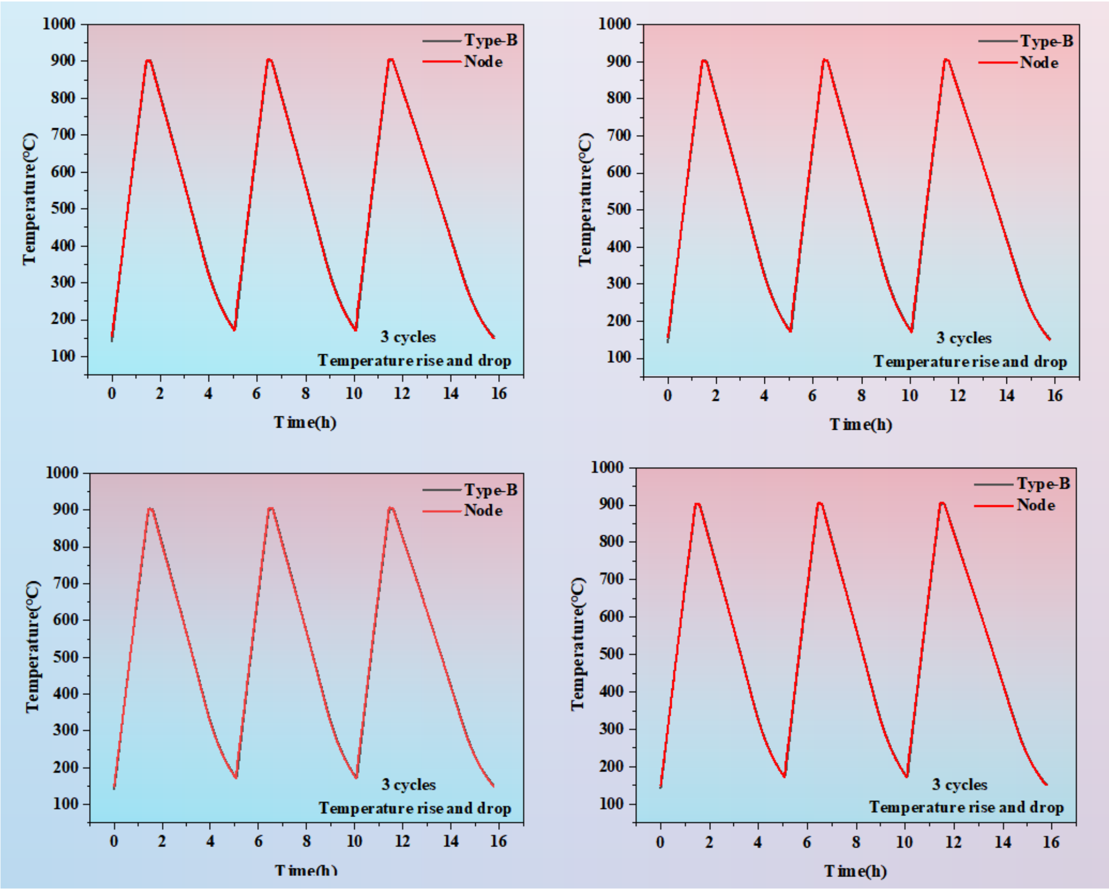

Figure S13. Result of cycle test in the array thermocouple.

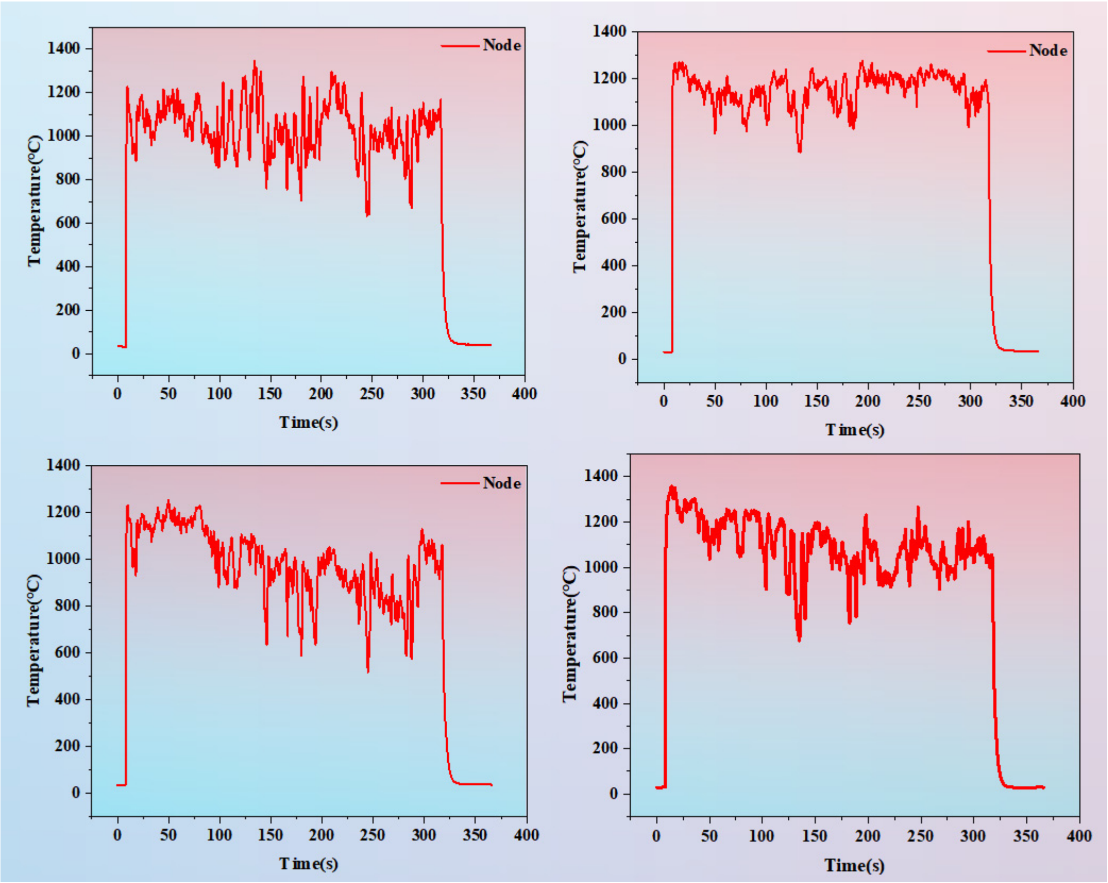

Figure S14. Result of high temperature flame in the array thermocouple.

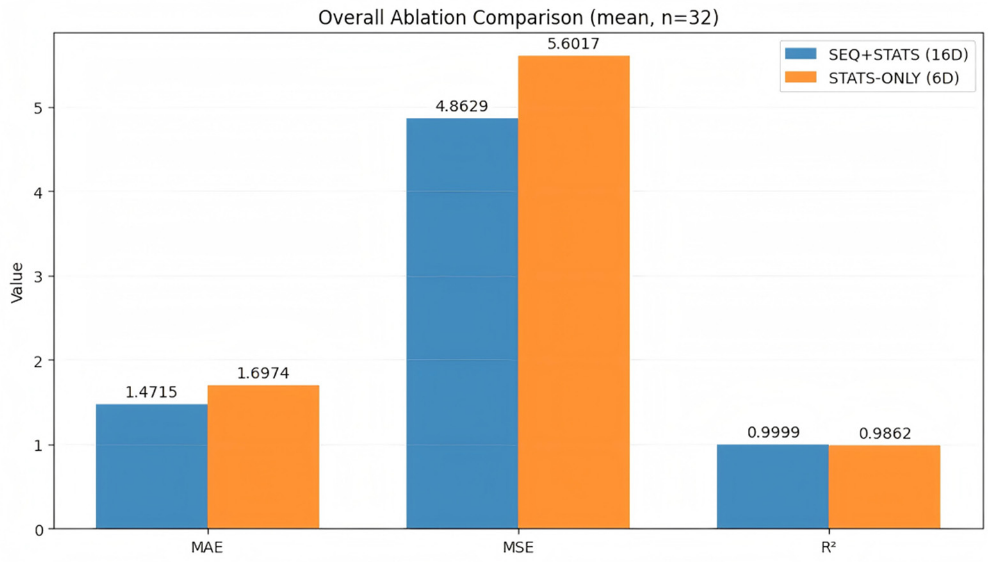

Figure S15. Overall Ablation Comparison.

Table S1. Deep Learning Model Architecture

Network Layer	Number of Neurons	Activation Function	Key Parameters
Input Layer	16	-	16(10-dimensional time-series features + 6-dimensional statistical features)
Hidden Layer 1	64	ReLU	Dropout Layer: Yes; Number of Neurons: 64
Hidden Layer 2	32	ReLU	Number of Neurons: 32; Activation Function: ReLU
Hidden Layer 3	16	ReLU	Number of Neurons: 16; Activation Function: ReLU
Output Layer	1	-	Output Dimension: 1 (calibrated temperature)
